# Peer Review of “Development of a Digital Platform to Promote Mother and Child Health in Underserved Areas of a Lower-Middle-Income Country: Mixed Methods Formative Study”

**DOI:** 10.2196/60430

**Published:** 2024-07-31

**Authors:** Jamie Sewan Johnston

**Affiliations:** 1 Stanford Center for Health Education, Stanford University, Stanford, CA, United States

**Keywords:** primary health care, mother and child health, community health worker, slums, digital applications, health communication

*This is the peer-review report for “Development of a Digital Platform to Promote Mother and Child Health in Underserved Areas of a Lower-Middle-Income Country: Mixed Methods Formative Study.”*

## Round 1 Review

### General Comments

This study [1] draws on multiple sources of data to assess the efficacy and feasibility of a video-based mobile health (mHealth) tablet intervention used to train and equip 10 community health workers (CHWs) in two slums in Pakistan. The overall strength of the paper is that the authors have collected in-depth qualitative data that can help inform the field on how to build and distribute such an intervention to improve the needs in low-resource communities. The paper should be strengthened by pinpointing the unique and new contributions of the study findings to inform the field on digital health education interventions for CHWs. While the intervention is described in detail, more work is needed to explicate why this study expands our understanding of digital health education for CHWs in deprived settings.

### Specific Comments

#### Major Comments

1. What the authors have found is largely expected and demonstrated in other work globally. Not surprisingly, they find that there is a severe lack of knowledge and critical need for education among CHWs and their patients to bring about behaviors that can improve maternal child health in low-resource communities. It is also not surprising that a well-resourced, highly supported, small-scale pilot (of just 10 CHWs trained) would be successful. While these findings are all important to describe in detail (as the authors have done), to provide more value in this field of work, I suggest the paper more explicitly emphasize what contribution the study adds to the literature. What are the new and important takeaways to improve how mHealth education interventions for CHWs are developed?
2. In addition to more explicitly pointing to the contributions of their findings, the paper could be strengthened considerably by a discussion of how their findings can inform how this small-scale pilot can be taken to scale effectively. The authors allude to this, but I think more could be added with regard to cost-effectiveness. It sounds like an expensive and involved intervention—to my understanding, providing a tablet to CHWs, hosting a two-day training, overseeing an apprentice week, and a refresher training, all on top of development of 14 videos and a calendar for patients. Information on the costs to develop and implement this intervention could be better described, and I would appreciate a critical lens on what would be needed to scale, including identification of barriers. I think the limitations they described with respect to CHW availability, connectivity, etc, should be folded into this discussion. I think this would set up well the ongoing work they describe to test real-world effectiveness in 250 mother-infant pairs.
3. A corollary to the above comment, because the intervention involves so many moving parts (ie, provision of a device, development of videos, in-person training and supervision), do their findings point to particular components of the intervention that are particularly important?
4. The introduction of the paper starts by highlighting the distinctions between the definitions of inequality, disparity, and inequity. I don’t think the comparison adds any value to the introduction. In fact, I was confused because, after the first paragraph, implications of an expensive mHealth intervention for equity are not discussed at all. Just because a study is conducted in a low-resource setting, does not mean that it is working to improve equity. If the authors want to focus on equity, I would appreciate a more critical lens on how the high costs of a digital intervention met with barriers like internet connectivity improve the situation of the poorest communities. (Otherwise, I suggest changing the introduction paragraph.) A video-based tablet intervention that relies upon internet could ostensibly be more effective in communities with more infrastructure and resources, and when scaled more widely in better resourced communities, digital interventions may actually broaden the gap between the haves and the have-nots. How can we think about ways digital interventions can be implemented to ensure this does not happen? (Is this why the in-person CHW-to-patient link is so important? Can this be unpacked?)
5. The phase two findings draw heavily upon the “qualitative feedback” obtained from CHWs and mothers about the Sehat Ghar application and tablet use, but there are scant details on how these data were collected and analyzed. If these qualitative findings are so prominent in the results and not merely anecdotal and complementary to other findings, they need to be described with the level of detail the preintervention in-depth interviews and focus group discussions were described. Were semistructured guides used? What framework was applied to the development of qualitative protocols? How were the data coded and analyzed? How many CHWs and mothers participated?
6. How were the two slums selected for focus? What inclusion/exclusion criteria, if any, were applied when thinking about geographic selection? How do the two slums generalize to the larger set of slums in Islamabad?
7. More of a description of the health systems in the slums is helpful for readers who are not familiar. How do CHWs fit into the larger health system? To my understanding, the 15 CHWs that were included in this study were completely new to the profession as “we identified volunteer women willing to become CHWs.” Why focus on completely new volunteers for the study rather than drawing upon the existing CHW workforce? Is it because there were not CHWs already at work in these areas? If there are other CHWs already serving these slums, can this be better described? Please also speak to the generalizability of the findings given that the CHWs in this study started with a much lower lack of knowledge given their novice status. If the study were to be done with experienced CHWs, perhaps the delta in knowledge gains would not be nearly as large.
8. A more detailed description of how participants (health care providers, CHWs, and mothers) were recruited for the study is needed. What was the sampling frame? What were the inclusion/exclusion criteria? What was the consent rate? What roles did the health care providers hold (ie, were they doctors, nurses, other roles)?
9. What were the protocols for conducting observations? Were they unannounced or were CHWs prepared in advance to know that the supervisors would be conducting the observations? Can you address limitations with respect to bias, as individuals generally behave quite differently when observed?
10. Do the authors have any analytics (ie, frequency of video views, engagement with the app) from the tablet/application that can be used to support the observation data and qualitative feedback on the intervention feasibility?

#### Minor Comments

1. In the Abstract, identify the larger geographic location of the communities.
2. Is there a more recent citation than the 2015 reference used for [2]?
3. The Methods section says that the initial five transcripts were coded independently by two members of the team. What about the remaining transcripts? Were there any checks/reconciliation on the coding of the remaining transcripts?
4. Consider moving some of the details of the intervention, including on page 7 of the Results, to the Methods section. When reading the Methods, I expected to see more of these details there and am a bit confused as to why they are included in the Results.
5. Suggest not paraphrasing Steve Jobs in the Discussion section.
6. The manuscript states that this pilot was conducted in 2018. The study also notes that ongoing work with 250 mother-infant pairs is currently being conducted, now 5 years later. Given how much has happened in the world, I am curious if the authors have any reflections on how the pandemic has changed the way we should understand and reflect their findings. (The pandemic need not be addressed in the manuscript, but the second to last paragraph of the Discussion talks about health emergencies. I am skeptical how such an involved pilot could be so quickly mobilized to respond to health emergencies. The authors should reflect on this if they believe findings point to this as a possibility. I also think the detailed statistics about flooding in Pakistan and other emergencies are out of scope for the paper. I don’t believe anyone needs convincing that health emergencies of this nature exist.)

## Round 2 Review

The authors have very thoughtfully and substantially revised the paper, making clear the methods and contributions of the study. I appreciate the detail with which the authors pointed to their edits in the revised manuscript and am satisfied with their changes.

At this point, I suggest only very minor revisions, asking authors to check grammar and conduct a copyedit of the paper. There are instances where a careful copyedit will improve the overall reading experience of the paper. For instance, in the Abstract, I suggest the following changes:

1. Drop the “the” in “Can the information-technology (IT) help these CHWs?”
2. Add a comma after “application” in “We explored answers through development and feasibility testing of Sehat Ghar, an android-based digital application to improve the communication capacity of volunteer CHWs in two slums of Islamabad.”
3. Do not capitalize “Focus Group Discussions” in the Methods section.

## Abbreviations

CHW: community health worker
mHealth: mobile health
